# Stereotactic Body Radiation Therapy (SBRT) for clinically localized prostate cancer: the Georgetown University experience

**DOI:** 10.1186/1748-717X-8-58

**Published:** 2013-03-13

**Authors:** Leonard N Chen, Simeng Suy, Sunghae Uhm, Eric K Oermann, Andrew W Ju, Viola Chen, Heather N Hanscom, Sarah Laing, Joy S Kim, Siyuan Lei, Gerald P Batipps, Keith Kowalczyk, Gaurav Bandi, John Pahira, Kevin G McGeagh, Brian T Collins, Pranay Krishnan, Nancy A Dawson, Kathryn L Taylor, Anatoly Dritschilo, John H Lynch, Sean P Collins

**Affiliations:** 1 Department of Radiation Medicine, Georgetown University Hospital, Washington, DC 20007, USA; 2 Department of Urology, Georgetown University Hospital, Washington, DC 20007, USA; 3 Department of Radiology, Georgetown University Hospital, Washington, DC 20007, USA; 4 Department of Oncology, Lombardi Comprehensive Cancer Center, Georgetown University Medical Center, Washington, DC 20007, USA

**Keywords:** Prostate cancer, SBRT, CyberKnife, SHIM, AUA, SF-12, Quality of life, Common Toxicity Criteria (CTC), Benign PSA bounce, Urinary symptom flare

## Abstract

**Background:**

Stereotactic body radiation therapy (SBRT) delivers fewer high-dose fractions of radiation which may be radiobiologically favorable to conventional low-dose fractions commonly used for prostate cancer radiotherapy. We report our early experience using SBRT for localized prostate cancer.

**Methods:**

Patients treated with SBRT from June 2008 to May 2010 at Georgetown University Hospital for localized prostate carcinoma, with or without the use of androgen deprivation therapy (ADT), were included in this retrospective review of data that was prospectively collected in an institutional database. Treatment was delivered using the CyberKnife® with doses of 35 Gy or 36.25 Gy in 5 fractions. Biochemical control was assessed using the Phoenix definition. Toxicities were recorded and scored using the CTCAE v.3. Quality of life was assessed before and after treatment using the Short Form-12 Health Survey (SF-12), the American Urological Association Symptom Score (AUA) and Sexual Health Inventory for Men (SHIM) questionnaires. Late urinary symptom flare was defined as an AUA score ≥ 15 with an increase of ≥ 5 points above baseline six months after the completion of SBRT.

**Results:**

One hundred patients (37 low-, 55 intermediate- and 8 high-risk according to the D’Amico classification) at a median age of 69 years (range, 48–90 years) received SBRT, with 11 patients receiving ADT. The median pre-treatment prostate-specific antigen (PSA) was 6.2 ng/ml (range, 1.9-31.6 ng/ml) and the median follow-up was 2.3 years (range, 1.4-3.5 years). At 2 years, median PSA decreased to 0.49 ng/ml (range, 0.1-1.9 ng/ml). Benign PSA bounce occurred in 31% of patients. There was one biochemical failure in a high-risk patient, yielding a two-year actuarial biochemical relapse free survival of 99%. The 2-year actuarial incidence rates of GI and GU toxicity ≥ grade 2 were 1% and 31%, respectively. A median baseline AUA symptom score of 8 significantly increased to 11 at 1 month (*p* = 0.001), however returned to baseline at 3 months (*p* = 0.60). Twenty one percent of patients experienced a late transient urinary symptom flare in the first two years following treatment. Of patients who were sexually potent prior to treatment, 79% maintained potency at 2 years post-treatment.

**Conclusions:**

SBRT for clinically localized prostate cancer was well tolerated, with an early biochemical response similar to other radiation therapy treatments. Benign PSA bounces were common. Late GI and GU toxicity rates were comparable to conventionally fractionated radiation therapy and brachytherapy. Late urinary symptom flares were observed but the majority resolved with conservative management. A high percentage of men who were potent prior to treatment remained potent two years following treatment.

## Background

For men with localized prostate cancer, the typical treatment with dose-escalated external beam radiation therapy (EBRT) involves fractionated radiation therapy using daily doses of 1.8-2.0 Gy for eight to nine weeks. Considering logistics and life responsibilities, such prolonged treatment courses present hardship for many patients. In addition, clinical data suggest that hypofractionated radiation therapy may be radiobiologically favorable to smaller fraction sizes in prostate cancer radiotherapy due to a potentially greater sensitivity of prostate cancer to larger daily radiation fractions [1]. Early data from trials of limited hypofractionation (fraction sizes from 2.5 to 3.5 Gy) revealed that such regimens are effective without undue toxicity [2]. Stereotactic body radiation therapy (SBRT) uses even larger daily fractions of radiation to take further advantage of this postulated radiobiological advantage. Early investigations of SBRT were performed with radiation delivery systems that did not allow continuous tracking of the prostate’s location with intrafractional adjustment of beam targeting if motion was detected [3]. Initially, the goal was to maintain a similar level of local control while sparing normal tissue using fairly low doses (33.5 Gy in five fractions). This relatively low biologically equivalent dose, perhaps in combination with geographic misses due to inadequate margins, led to relatively poor biochemical control [3].

CyberKnife® (Accuray Incorporated, Sunnyvale, CA) delivers hundreds of individualized circular beams with a targeting error of less than 1 mm allowing the safe delivery of highly conformal treatment plans with steep dose gradients [4]. Unlike standard image-guided radiation therapy (IGRT), the CyberKnife system incorporates a real-time tracking system that provides updated prostate position information to the robot to correct the targeting of the therapeutic beam during treatment [5]. This feature allows for a reduction in the planning target volume (PTV) and, therefore, better limits the dose to surrounding critical organs. Such technology has enabled other institutions to administer SBRT to the prostate (doses of 35–36.25 Gy) with excellent biochemical disease-free survival yet with toxicities similar to conventional treatments [6-8]. Here we present our early institutional experience with SBRT for clinically localized prostate cancer.

## Methods

### Patient selection

Patients eligible for study inclusion had histologically-confirmed adenocarcinoma of the prostate treated per our institutional protocol. Exclusion criteria included clinical stage T3, involved lymph nodes or distant metastases on imaging and/or prior pelvic radiotherapy. Institutional IRB approval was obtained for retrospective review of data that was prospectively collected in our institutional database.

### SBRT treatment planning and delivery

Four gold fiducials were placed into the prostate. Seven days after fiducial placement, patients underwent MR imaging followed shortly thereafter by a thin-cut CT scan. Fused CT and MR images were used for treatment planning. The clinical target volume (CTV) included the prostate and the proximal seminal vesicles (to the point where the seminal vesicles separate). The PTV equaled the CTV expanded 3 mm posteriorly and 5 mm in all other dimensions. The prescription dose was 35–36.25 Gy to the PTV delivered in five fractions of 7–7.25 Gy corresponds to a tumor EQD2 of approximately 85–90 Gy assuming an α/β ratio of 1.5. In general, older patients with poor baseline urinary function were treated with 35 Gy.

**Figure 1:**
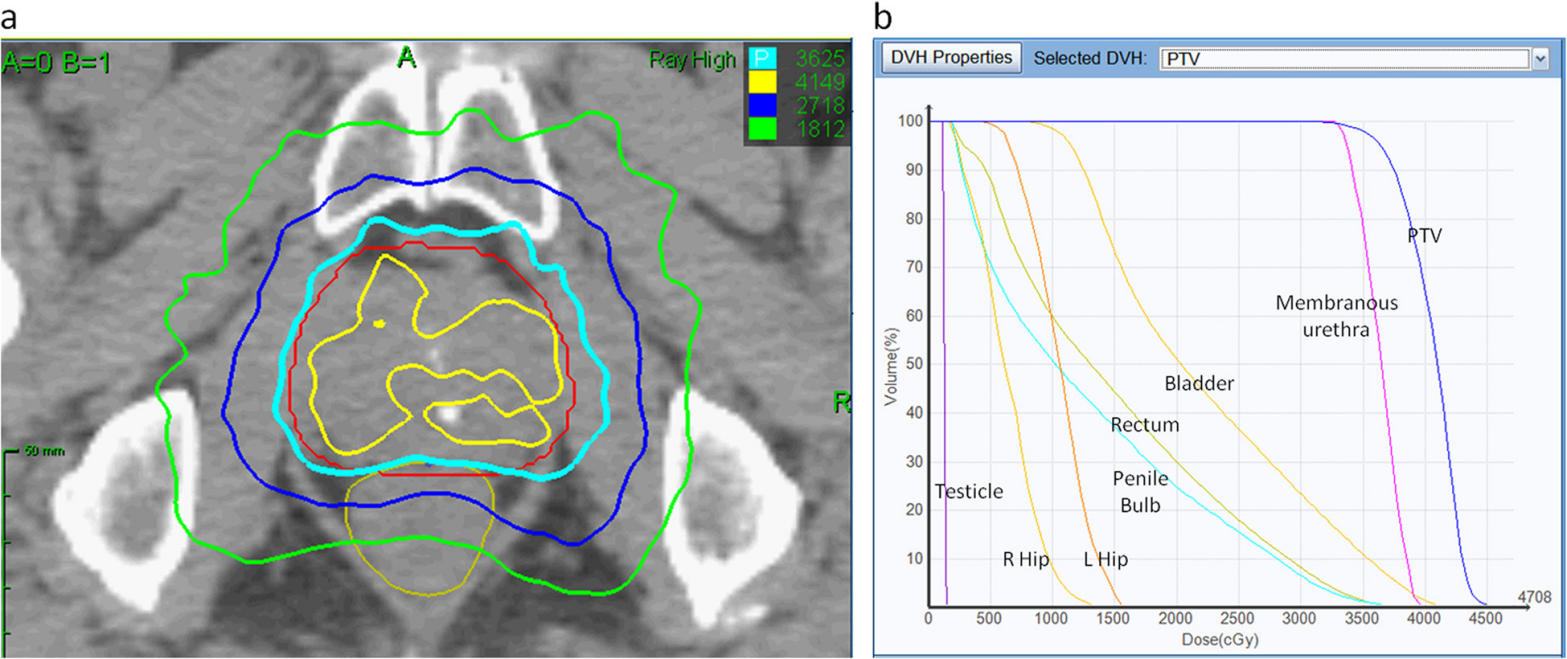
**(a) Treatment planning axial computed tomography images demonstrating the prostate (red line) and rectum (brown line).** Isodose lines shown as follows: 115% of the prescription dose, yellow line; 100% of the prescription dose, light blue line: 75% of the prescription dose, dark blue line; and 50% of the prescription dose, green line. (**b**) A typical dose–volume histogram for CyberKnife treatment of a prostate cancer patient.

Treatment plans were composed of hundreds of pencil beams using one to two circular collimator to generate highly conformal plans (mean new conformity index of 1.28 [range, 1.12-1.59]). Plans were inhomogeneous by design (mean homogeneity index of 1.29 [range, 1.23-1.42]) to minimize dose to adjacent critical structures (Figure 1a). However, the prescription isodose line was limited to ≥ 75% to restrict the maximum prostatic urethra dose to 133% of the prescription dose. The rectum, bladder, testes, penile bulb and membranous urethra were contoured and evaluated with dose-volume histogram analysis during treatment planning using Multiplan (Accuray Inc., Sunnyvale, CA) inverse treatment planning. A typical dose-volume histogram is shown in Figure 1b and critical structure dose constraints are shown in Table 1. To minimize the risk of local recurrence, no attempt was made to limit the dose to the prostatic urethra or neurovascular bundles [9,10]. Radiation was delivered every other day to a mean prescription isodose line of 77% (range, 75-81%) in 5 approximately 1 hour long treatments, with a mean treatment duration of 10.6 days (range, 5–16 days). On average, 247 beams were employed (range, 199–289 beams) to treat the mean prescription volume of 135 cc (range, 61–258 cc) with mean percent target coverage of 95.20% (range, 93.65-96.95%). Target position was verified every 30–60 seconds during treatment using paired, orthogonal kV images (total imaging effective dose equals 17.5 mSv).

**Table 1:** Dose targets and constraints for treatment planning

**36.25 Gy plan constraints**	
**Global Max Dose**	**48.33 Gy**
**PTV**	**V (36.25 Gy) ≥ 95%**
**Rectum**	**V (36 Gy) < 1 cc**
	**V (100%) < 5%**
	**V (90%) < 10%**
	**V (80%) < 20%**
	**V (75%) < 25%**
	**V (50%) < 50%**
**Bladder**	**V (37 Gy) < 5 cc**
	**V (100%) < 10%**
	**V (50%) < 40%**
**Penile Bulb**	**V (29.5 Gy) < 50%**
**Membranous urethra**	**V (37 Gy) < 50%**
**Sigmoid colon**	**V (30 Gy) < 1 cc**
**Testicles**	**D (20%) < 2 Gy**

### Follow-up and statistical analysis

Prostate-specific antigen (PSA) and total testosterone levels were obtained before treatment, one month after the completion of SBRT, and during routine follow-up visits every 3 months for the first year and every six months for the second year of follow-up. Alpha-antagonist, antidiarrheal and phosphodiesterase type 5 (PDE5) inhibitor utilization was documented at each visit.

Toxicity was prospectively documented at follow-up visits using the National Cancer Institute (NCI) Common Toxicity Criteria (CTC) version 3.0. Acute toxicity was defined as experiencing toxicity during or within 6 months of radiation therapy. Late toxicity was defined as occurring at least 6 months after delivery of radiation therapy. Transient and chronic late toxicities were included. The genitourinary toxicities analyzed were hematuria, dysuria, incontinence, urinary urgency/frequency and retention. The gastrointestinal toxicities analyzed were bowel frequency/urgency, proctitis and rectal bleeding. In general, Grade 1 toxicity represents minimal side effect not requiring medications for symptom control. Pre-treatment symptoms were counted as Grade 1 toxicity if they increased in severity. Grade 2 toxicity indicates symptoms requiring new medication (i.e. alpha-antagonist or antidiarrheal) or increase in dose of previously prescribed medication. Grade 3 indicates complications requiring minor surgical intervention (i.e., transurethral resection or laser coagulation). At each follow-up visit, toxicity events were scored independently for each of the different toxicity types and the highest GU and GI toxicity was determined for each patient.

Quality of life (QOL) was assessed before and after treatment using the Short Form-12 Health Survey (SF-12), the American Urological Association Symptom Score (AUA) and Sexual Health Inventory for Men (SHIM) questionnaires. The Medical Outcomes Study Short Form-12 Health Survey (SF-12) [11], which contains two subscales, the Mental Component Summary (MCS) and the Physical Component Summary (PCS), with higher values indicating better quality of life. AUA scores range from 0–35 with higher values representing worsening urinary symptoms [12]. SHIM scores range from 0–25 with lower values representing worsening sexual symptoms [13].

Student’s *t*-test and chi-square analysis were used to assess differences in ongoing PSA, testosterone and quality of life scores (SF-12, AUA and SHIM) in comparison to baseline. Sample medians and ranges were used to describe continuous variables including PSA and testosterone. Based upon published results, a benign PSA bounce was defined as a PSA rise of 0.2 ng/mL or more above its previous nadir with a subsequent decline to that nadir or lower [14]. Actuarial likelihood estimates for late toxicities were determined using the Kaplan-Meier method. The highest GU or GI toxicity available for each patient was evaluated for the actuarial analysis. SF-12 analysis with norm-based scores (Mean = 50, standard deviation = 10) [15] was performed using IBM SPSS Statistics 20. The minimally important difference (MID) in AUA score was defined as a change of one-half standard deviation (SD) from the baseline [16]. As previously reported, late urinary symptom flare was defined as an increase of ≥ 5 points above baseline with a degree of severity in the moderate to severe range (AUA score ≥ 15) [17]. The flare was considered resolved when either the AUA score dropped to < 15 or the score returned to < 5 points above the patient’s pretreatment baseline. Erectile dysfunction (ED) was categorized into five categories of severity based on the patient’s SHIM score: no ED (22–25), mild ED (17–21), mild to moderate (12–16), moderate (8–11) and severe (1–7) [18]. Patients were considered potent if they scored ≥ 10 on the SHIM [18]. To limit the effect of attrition bias, statistical analysis was limited to time points in which ≥ 80% of the patient data were available.

## Results

From June 2008 to May 2010, 100 prostate cancer patients were treated per our institutional SBRT monotherapy protocol (Table 2). They were ethnically diverse with 57% being of Caucasian ancestry and a median age of 69 years (range, 48–90 years). By D’Amico classification, 37 patients were low-, 55 intermediate-, and 8 high-risk. Eleven patients also received androgen deprivation therapy (ADT). Most received short term ADT (three to six months), while two high risk patients received long term ADT (two to three years). Eighty-five percent of the patients were treated with 36.25 Gy in five 7.25 Gy fractions.

**Table 2:** Patient characteristics and treatment

**Age (yrs)**	**Percent Patients (n = 100)**
< 60	8
60-69	45
70-79	43
≥ 80	4
Race	
White	56
Black	37
Hispanic	5
Asian	2
Pre-Tx PSA (ng/ml)	
≤ 10	87
> 10 and ≤ 20	12
> 20	1
T Stage	
T1b	1
T1c	75
T2a	12
T2b	8
T2c	4
Gleason Score	
2 + 3 = 5	2
3 + 2 = 5	1
3 + 3 = 6	42
3 + 4 = 7	40
4 + 3 = 7	10
3 + 5 = 8	1
4 + 4 = 8	4
Risk Groups	
Low Risk	37
Intermediate Risk	55
High Risk	8
Hormone Treatment	
Yes	11
No	89
Dose (Gy)	
35.00	15
36.25	85

**Figure 2:**
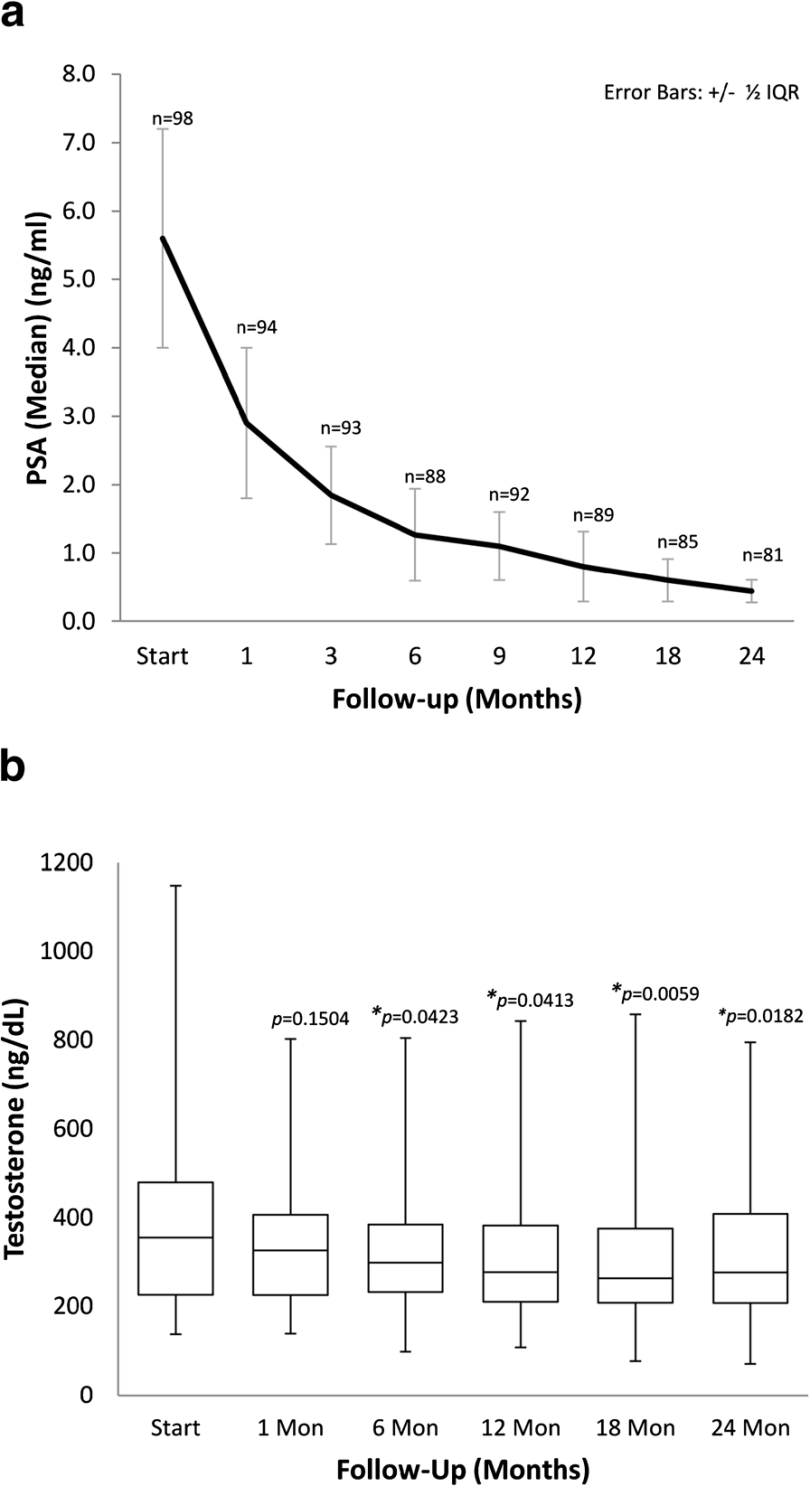
**Pre- and post-treatment PSA and testosterone levels: (a). PSA levels for all patients (error bars indicate interquartile intervals) and (b). Box-and-Whisker plot of total testosterone levels.** Only patients who did not receive androgen deprivation therapy (ADT) were included in the testosterone diagram. The p values were from *χ*^2^-analysis with baseline testosterone levels.

The median follow-up was 2.3 years (range, 1.4-3.5 years). At two years post-treatment, the median pre-treatment PSA of 6.2 ng/ml (range, 1.9-31.6 ng/ml) declined to a median of 0.49 ng/ml (range, 0.1-1.9 ng/ml) (Figure 2a). Benign PSA bounces occurred in 31% of patients with a median PSA bounce of 0.5 ng/ml (range, 0.2-2.2 ng/ml) and the median time following treatment to the PSA bounce was 15 months (range, 3–21 months). There was one biochemical failure, occurring in a high-risk patient. Prostate biopsy confirmed local recurrence and ADT was initiated. The overall two-year actuarial biochemical relapse free survival was 99%.

Pre-treatment total serum testosterone levels ranged from 4.75 nmol/L to 39.84 nmol/L with a median value of 12.29 nmol/L. Twenty-nine percent of patients were hypogonadal (total serum testosterone level below 8 nmol/L) prior to SBRT. At two years the median serum testosterone value of 9.78 nmol/L (range, 2.46- 27.60 nmol/L) was significantly lower than the pre-treatment value (*p* < 0.02, Figure 2b). The median absolute reduction was small (2.5 nmol/L) and the median percent reduction was 20.5%. The pre-treatment and 2-year biochemical hypogonadism rates were not statistically significantly different (data not shown).

**Figure 3:**
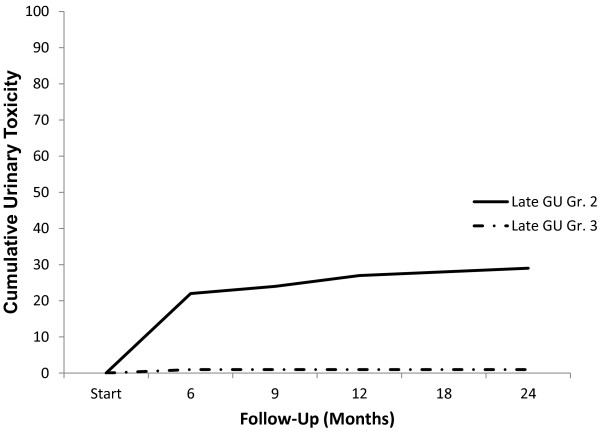
Cumulative late urinary toxicity (grades 2 and 3).

**Figure 4:**
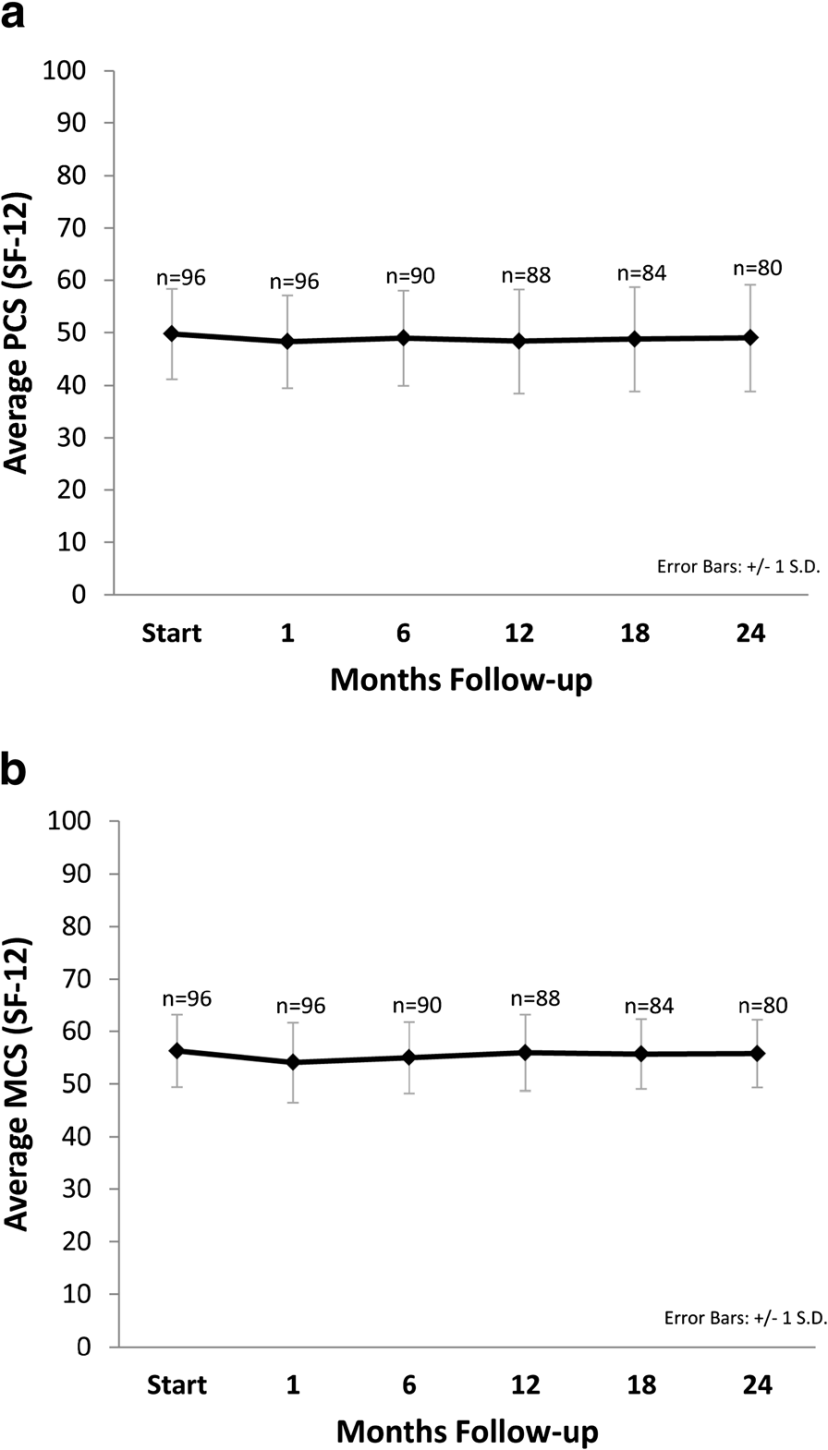
**Short Form-12 (SF-12) Health Survey quality of life: (a) SF-12 physical component score (PCS) and (b) SF-12 mental component score (MCS).** The graphs show unadjusted changes in average scores over time. The scores range from 0–100 with higher values representing improved health status. Numbers above each time point indicate the number of observations contributing to the average.

The prevalence of GU and GI toxicities following treatment is shown in Table 3. The prevalence of single symptoms as well as the highest GI and GU toxicity per patient are depicted independently for each follow-up visit. Acute urinary grade 2 toxicities requiring alpha-antagonists occurred in 35% of patients (Table 3). Acute bowel frequency and/or spasms requiring anti-diarrheals were uncommon (5%). The 2-year actuarial incidence rates of late GI and GU toxicity ≥ grade 2 were 1% and 31%, respectively. Actuarial incidence rates of late grade 2 and 3 GU toxicities are demonstrated in Figure 3. Grade 3 toxicities rates were low with one case of hematuria requiring transurethral resection of the prostate (TURP). The patient’s prostatic volume was 85 grams and his pretreatment AUA score was 18. At two year post-treatment, the patients' perceptions of their physical health (Figure 4a) and mental health (Figure 4b) were not statistically different from baseline (*p* = 0.76 and 0.90, respectively).

**Table 3:** Prevalence of CTC graded gastrointestinal (GI) and genitourinary (GU) toxicities at each follow-up

**Follow-up (months)**		**1**	**3**	**6**	**9**	**12**	**18**	**24**
**Toxicity**	**Grade**	**%**	**%**	**%**	**%**	**%**	**%**	**%**
Bowel Frequency/Urgency	0	72	83	78	81	91	95	93
	1	23	16	20	18	8	5	7
	2	5	1	1	1	1	0	0
Proctitis	0	86	97	95	95	99	99	100
	1	14	3	5	5	1	1	0
	2	0	0	0	0	0	0	0
Rectal Bleeding	0	94	98	96	95	98	93	96
	1	6	2	4	5	2	7	4
	2	0	0	0	0	0	0	0
Highest GI	0	60	79	71	73	87	89	89
	1	35	20	28	26	12	11	11
	2	5	1	1	1	1	0	0
Hematuria	0	96	97	99	96	97	98	94
	1	4	2	0	3	2	1	4
	2	0	1	0	0	0	0	0
	3	0	0	1	1	1	1	1
Dysuria	0	71	91	97	88	93	96	93
	1	29	9	3	13	7	4	7
	2	0	0	0	0	0	0	0
Incontinence	0	85	86	86	84	89	89	88
	1	13	14	14	16	11	10	12
	2	2	0	0	0	0	1	0
Urinary Frequency/Urgency	0	81	90	90	93	93	90	88
	1	19	10	9	7	6	8	11
	2	0	0	1	0	1	2	1
Retention	0	55	64	63	70	75	70	76
	1	10	11	14	11	9	11	9
	2	35	24	23	19	16	19	16
Highest GU	0	28	52	53	54	59	53	57
	1	36	23	23	26	23	26	26
	2	35	25	23	19	17	20	17
	3	0	0	1	1	1	1	1

**Figure 5:**
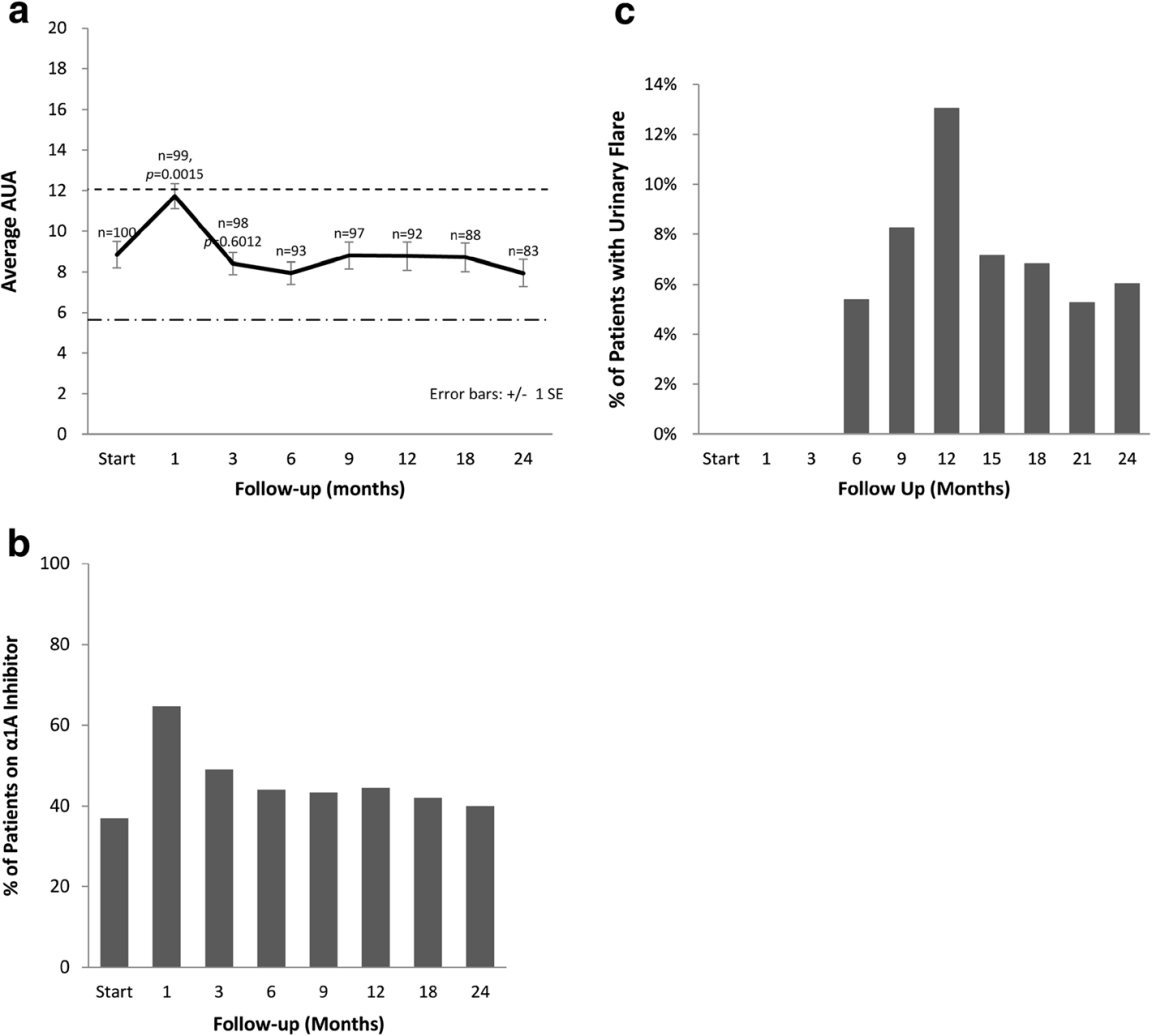
**Urinary quality of life: (a) AUA score, (b) alpha antagonist utilization and (c) urinary symptom flare.** The graphs show unadjusted changes in average scores over time for each domain. AUA scores range from 0–35 with higher values representing worsening urinary symptoms. Numbers above each time point indicate the number of observations contributing to the average. The thresholds for clinically significant changes in scores (½ standard deviation above and below the baseline) are marked with dashed lines. Error bars indicate 95% confidence intervals.

The majority of patients had mild to moderate lower urinary tract symptoms prior to treatment with a mean baseline AUA of 8 (range, 0–24) (Table 4). At one month post-treatment the mean AUA symptom score increased to 11, returning to baseline at 3 months (*p* = 0.60, Figure 5a). This increase was statistically significant (*p* = 0.001) but of borderline clinical significance (MID = 3.22). Alpha-antagonist utilization peaked at one month post-treatment at 65% patient utilization then slowly decreased to near baseline, with 40% of patients reporting use at two years (Figure 5b). Transient late urinary symptom flare (≥ 6 months after completing treatment) occurred in 21% of the patients (Figure 5c). The median flare magnitude was 9 (range, 5–22), the median time to flare was 9 months (range, 6–21 months), and the median duration of flare was 3 months (range, 3–9 months). Twenty-eight percent of the flares lasted longer than six months.

**Table 4:** Pre-treatment Quality of Life (QOL) scores

Baseline SF-12 Score	Pretreatment Score	
PCS^*^(n = 96)	48.9 (19.7-60.8)	[*SD* = 8.56]
MCS^*^(n = 96)	54.5 (26.2-63.9)	[*SD* = 6.88]
Baseline AUA Score	% Patients (n = 100)	
0-7 (mild)	46	
8-19 (moderate)	47	
≥ 20 (severe)	7	
Baseline SHIM Score	% Patients (n = 98)	
< 8 (Severe ED)	37	
8-11 (Moderate ED)	2	
12-16 (Mild-Moderate ED)	16	
17-21 (Mild ED)	27	
22-25 (no ED)	18	

^*^ Norm-based scoring for SF-12 with population mean = 50 and SD = 10.

Abbreviations: *SF-12*, Short-form-12; *PCS*, physical component score; *MCS*, mental component score; *SD*, standard deviation; *AUA*, American urological association; *SHIM*, sexual health inventory for men; *ED*, erectile dysfunction.

**Figure 6:**
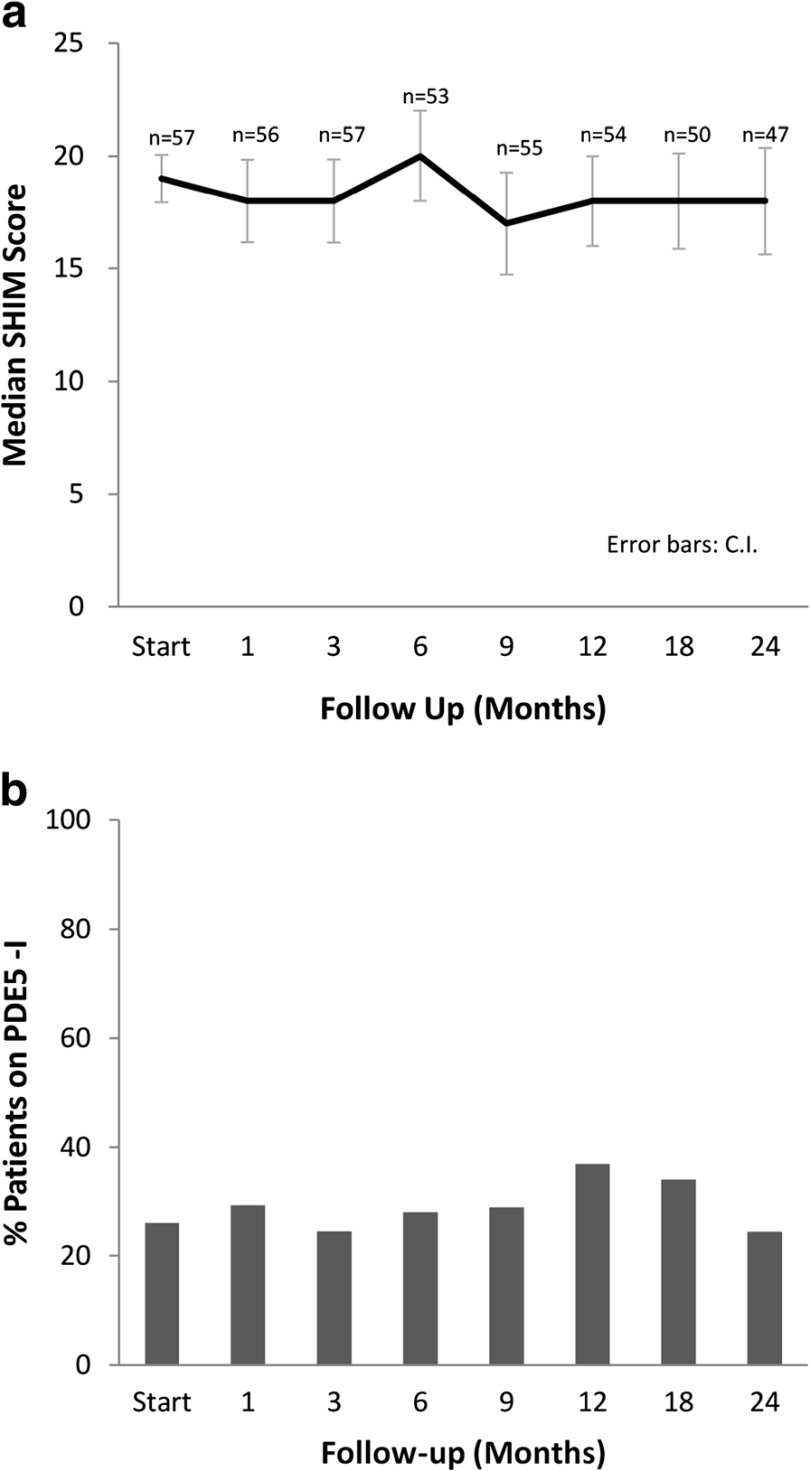
**Sexual quality of life: (a) SHIM and (b) PDE5 utilization.** The graphs show unadjusted changes in average scores over time for each domain among men who were potent at baseline (N = 57). SHIM scores range from 0–25 with lower values representing worsening sexual symptoms. Numbers above each time point indicate the number of observations contributing to the average.

Prior to treatment, a significant portion of patients had erectile dysfunction based upon the SHIM (Table 4). We limited our sexual function analysis to the 57 patients who had a pretreatment SHIM score ≥ 10 and who did not receive ADT. At two years post-treatment, the median SHIM decreased from a baseline of 19 to 18 (*p* = 0.003, Figure 6a). There was no statistically significant change in PDE5 inhibitor utilization over time (Figure 6b). At 2-years post-treatment, 51% of patients had utilized a PDE5 inhibitor at some point during follow up. Seventy-nine percent of these patients maintained potency (as defined as a SHIM of ≥10) at two year post-treatment (Table 5). The decline in potency at two years was unlikely due to aging, as the average age of potent patients was not statistically different from non-potent patients (*p* = 0.41).

**Table 5:** SHIM scores at baseline and following treatment

	**Pre-Tx**	**1 Mon**	**3 Mon**	**6 Mon**	**9 Mon**	**12 Mon**	**18 Mon**	**24 Mon**
**(Total N)**	**(57)**	**(56)**	**(57)**	**(53)**	**(55)**	**(54)**	**(50)**	**(47)**
**% Potent** (SHIM ≥ 10)	**100.0%**	**85.7%**	**78.9%**	**83.0%**	**67.3%**	**77.8%**	**76.0%**	**78.7%**
**SHIM 22 ~ 25** (No ED)	28.1%	26.0%	22.2%	28.2%	22.0%	24.5%	22.8%	28.5%
**SHIM 17 ~ 21** (Mild ED)	43.9%	27.6%	24.9%	25.1%	15.9%	20.2%	16.7%	16.7%
**SHIM 12 ~ 16** (Mild-Moderate ED)	24.6%	12.2%	15.2%	12.5%	4.9%	10.1%	12.2%	8.4%
**SHIM 8 ~ 11** (Moderate ED)	3.5%	9.2%	2.8%	6.3%	6.1%	7.2%	7.6%	8.4%
**SHIM < 8** (Severe ED)	0.0%	10.7%	13.9%	11.0%	18.3%	15.8%	16.7%	16.7%

Abbreviations: *Pre-Tx*, pre-treatment; *SHIM*, sexual health inventory for men; *ED*, erectile dysfunction.

## Discussion

While IMRT and brachytherapy are the most commonly used radiation therapy modalities for clinically localized prostate cancer, SBRT utilization is increasing [19]. The accuracy assured by intra-fraction image guidance, which allows the use of smaller CTV-PTV margins, may allow safe prostate treatment in four or five large (e.g., 7–9.5 Gy) radiation fractions. Emerging data from single institutional series [6-8] and a small multi-institutional Phase I study [20] suggest that this approach may provide similar clinical outcomes as other radiation modalities with high rates of biochemical control and low rates of grade 3 and higher toxicities. A recent update of grouped series confirmed SBRT achieved 5-year biochemical disease-free survival of 93% in patients with favorable prognosis [21]. Based on these reports, as well as patient preference for a shorter treatment course, SBRT utilization is likely to continue to increase.

Our institutional experience adds to the growing body of evidence supporting the effectiveness and safety of SBRT. Our early PSA outcomes have been favorable. The two-year post-treatment PSA nadir of 0.49 ng/mL predicts a high rate of long-term disease control [22]. This PSA response is unlikely due to declines in testosterone since the majority of patients had stable testosterone over time. As with other SBRT series [6,7,20] and other radiation therapy modalities [23,24], benign PSA bounces were common and transient. Considering that our series includes a higher percentage of intermediate- and high-risk patients than others [6-8,20], our 99% actuarial 2-year biochemical failure-free survival rate is reassuring.

There is limited data on the use of SBRT for unfavorable patients (6). To date, SBRT studies have included mostly favorable patients due to the concern over limited coverage of potential extracapsular extension and seminal vesicle invasion. A recent dosimetric study suggest that SBRT delivers adequate dose to areas of potential extracapsular extension and the proximal seminal vesicles in unfavorable patients [25]. Although prospective studies are needed to confirm long-term tumor control, currently available data are comparable to results reported for brachytherapy and conventional external beam radiotherapy [26] .

Toxicity following SBRT was similar to that following external beam radiation therapy or brachytherapy [27]. Late Grade 2 and Grade 3 GU toxicity were observed in 30% and 1% of patients, respectively (Figure 3). Alpha antagonist utilization was the most common grade 2 toxicity, but similar to brachytherapy treatment, it returned to baseline one to two years post-treatment [28]. To maximize patient comfort, it is currently our institutional policy to initiate prophylactic alpha antagonist use prior to initiating treatment. The single grade 3 toxicity was hematuria requiring a TURP. This patient had a history of benign prostatic hypertrophy with a large prostate and two prior TURP procedures and was dependent on intermittent catheterization prior to receiving SBRT. As others have, we recommend that urethral instrumentation following treatment should be limited in patients treated with SBRT [7]. The SF-12 scores showed no significant change throughout the follow-up period suggesting that toxicities did not significantly adversely affect the patients' perceptions of their health.

As seen by other SBRT series [20], our mean AUA scores returned to baseline by three months post treatment. However, a minority of patients experienced a clinically meaningful increase in their urinary symptoms greater than six months after the completion of treatment. To our knowledge this is the first reported series to characterize late urinary symptom flare [17,29-32] in patients receiving SBRT. Like conventionally fractionated external beam radiation therapy and brachytherapy, late urinary toxicity occurred in a minority of our patients and resolved with conservative management (urinary anesthetics, alpha-blockers and/or brief steroid tapers). Endoscopic evaluation of these patients has suggested that this may be caused by urethritis (unpublished data). Knowledge of these late urinary toxicities and their resolution with conservative management will enable clinicians to relieve patient concerns and prevent unnecessary invasive procedures such as cystoscopy and/or TURP.

In our opinion, late urinary symptom flare may be exacerbated by the high central doses in our relatively inhomogeneous plans (Figure 1a). With the aim of reducing urinary symptoms, we have modified our institutional protocol. Specifically, we have reduced the anterior/superior PTV expansion to 3 mm to reduce the bladder neck dose. In addition, it is now our practice to prescribe to ≥ 80% isodose line to reduce the dose delivered to the prostatic urethra. Finally, to further reduce the prostatic urethral dose, we have restricted the maximum prostatic urethral dose to 110% of the prescription dose. We believe that such modifications will reduce the incidence and severity of late urinary symptom flare without increasing the risk of local failures [9,10].

As in other radiation therapy series, our patients were elderly with poor baseline erectile function and high PDE5 inhibitor utilization prior to treatment [33-35]. Similar to other SBRT series [8,36], 79% of patients who were sexually potent prior to treatment maintained potency at two years’ post-treatment. These results are comparable to results with conventionally fractionated EBRT or brachytherapy [37,38]. Interestingly, as observed by others, the greatest decline in SHIM occurred in the first year with sexual function stabilization afterwards [39]. The etiology of this early decline in SHIM is uncertain. Fifty-one percent of patients utilized PDE5 inhibitors in follow-up. It is not clear why utilization was not higher following treatment. Potential explanations include patient indifference [33], limited effectiveness [40] or the high cost of such medications.

Our study should be examined in the context of the study design. Our study is limited by the retrospective nature of the analysis. However, subjects were accrued consecutively and all data were collected prospectively in a centralized database, therefore limiting selection and reporting bias present in pure retrospective studies. In addition, the narrow focus of the AUA on obstructive symptoms and SHIM on erectile function is another limitation of our study [41]. Future studies should employ more comprehensive instruments to assess the effect of prostate SBRT on overall urinary and sexual function in this elderly patient population.

## Conclusions

SBRT was well-tolerated for these patients with clinically localized prostate cancer. Early PSA results suggest a biochemical response similar to other standard radiation therapy options. Benign PSA bounces were common. Rates of late GI and GU toxicity are comparable to conventionally fractionated radiation therapy and brachytherapy. Late urinary symptom flares are observed but the majority resolved with conservative management. A high percentage of men who were potent prior to treatment remained potent two years following treatment.

## Abbreviations

ADT: Androgen deprivation therapy; AUA: American urological association; CTC: Common toxicity criteria; CTV: Clinical target volume; DVH: Dose-volume histogram; GI: Gastrointestinal; GU: Genitourinary; GTV: Gross target volume; PTV: Planning target volume; QoL: Quality of life; SHIM: Sexual health inventory for men; SBRT: Stereotactic body radiation therapy; SF-12: Short Form-12 health survey.

## Competing interests

SP Collins and BT Collins serve as clinical consultants to Accuray Inc. The Department of Radiation Medicine at Georgetown University Hospital receives a grant from Accuray to support a research coordinator. The other authors declare that they have no competing interests.

## Authors’ contributions

LC and SS are lead authors, who participated in data collection, data analysis, manuscript drafting, table/figure creation and manuscript revision. SU aided in the quality of life data collection and maintained the patient database. EO aided in the quality of life data collection and statistical analysis. AJ aided in the quality of life data collection. VC aided in the quality of life data collection. HH aided in the quality of life data collection and maintained the patient database. SL aided in the quality of life data collection. JK aided in the quality of life data collection and maintained the patient database, aided in data collection, and participated in initial data interpretation. SL is the dosimetrist who developed the majority of patients’ treatment plans, and contributed to the dosimetric data analysis and interpretation. GP, KK, GB, JP, KM, aided in clinical data collection. BC, PK, ND, participated in the design and coordination of the study. KT aided in statistical analysis, quality of life analysis and manuscript revision. AD is a senior author who aided in drafting the manuscript. JL is a senior author who aided in drafting the manuscript. SC was the principal investigator who initially developed the concept of the study and the design, aided in data collection, drafted and revised the manuscript. All authors read and approved the final manuscript.
